# Imaging biobanks: operational limits, medical-legal and ethical reflections

**DOI:** 10.3389/fdgth.2024.1408619

**Published:** 2024-08-29

**Authors:** Emanuele Capasso, Claudia Casella, Mariagrazia Marisei, Mario Tortora, Francesco Briganti, Pierpaolo Di Lorenzo

**Affiliations:** Department of Advanced Biomedical Sciences, University of Naples Federico II, Naples, Italy

**Keywords:** imaging biobanks, holistic biobanks, big data, legal framework, personalized medicine, digital health

## Abstract

The extraordinary growth of health technologies has determined an increasing interest in biobanks that represent a unique wealth for research, experimentation, and validation of new therapies. “Human” biobanks are repositories of various types of human biological samples. Through years the paradigm has shifted from spontaneous collections of biological material all over the world to institutional, organized, and well-structured forms. Imaging biobanks represent a novel field and are defined by European Society of Radiology as: “organized databases of medical images, and associated imaging biomarkers shared among multiple researchers, linked to other biorepositories”. Modern radiology and nuclear medicine can provide multiple imaging biomarkers, that express the phenotype related to certain diseases, especially in oncology. Imaging biobanks, not a mere catalogue of bioimages associated to clinical data, involve advanced computer technologies to implement the emergent field of radiomics and radiogenomics. Since Europe hosts most of the biobanks, juridical and ethical framework, with a specific referral to Italy, is analyzed. Linking imaging biobanks to traditional ones appears to be a crucial step that needs to be driven by medical imaging community under clear juridical and ethical guidelines.

## 1 Introduction

Even though the term “biobank” originally arose in 1996 (1), a scientific work on its definition wasn't published until 2013 (2). This indicates that although terminology like “biobank” occasionally crop up in contemporary usage, there wasn't much scientific consensus or knowledge regarding their application. A human biobank is an assortment of human biological specimens, such as cells, tissue, blood, and DNA, along with associated data and additional biomolecular resources that can be employed in medical research (3). The remarkable development of health technology has led to a rise in interest in biobanks, which offer a special wealth of resources for study, testing, and validation of novel treatments. The paradigm of spontaneous collecting has changed over time (4), going from spontaneous biological material collections all over the world, thanks to the donations of patients and their families aimed at the development of research to institutional, organized and structured according to common rules shared (biobanks): an important research tool whose positive results they bring benefits not only to the donor and his family but also to the whole human community (5).

Paskal et al. (6) provide an overview of Europe's biobanks and the global network. There is a lot of variety in the official definitions of the term “biobank” (2). According to Hewitt and Watson's proposed definition, which was developed following a survey of sample management enthusiasts, a biobank is defined as a facility that collects, preserves, stores, and distributes biological samples along with related data. It adheres to standard operating procedures and offers material for use in research and clinical settings. The International Society for Biological and Environmental Repositories defines “Biobank” as a synonymous of “Repository”, that is: “In its simplest form, a place where collected parts or the whole of organisms and/or environmental specimens are stored for safekeeping. In the context of this document, the term applies more extensively to any entity focused on management and operations of specimens and associated data primarily intended for research purposes. Alternative terms may include biobank, biorepository, biological resource center, collection (e.g., microbial collection center, data collection center), cryogenic biobank, digital repository, gene bank, biodiversity biobank seed bank, virtual biobank, veterinary biobank, culture collection, gene bank, environmental specimen bank, tissue bank, cell bank among many others” (7). Human biobanks play a key role in all the characteristics of customized medicine. The so-called “P4”, which consists of four properties (8, 9), defines a novel method for a personalized medicine, that represents a tailored medical model that aims to provide prevention and treatment strategies based on an individual genetic profile. The goal of this innovative approach to the patient and health care is to determine which treatments or methods are most likely to work for a given patient based on their biological traits and the group to which they belong.

The four qualities that make up the"P4” paradigm are:

1. Predictive: the capacity to quickly, accurately, and broadly assess risk for particular diseases using procedures that are inexpensive and easy to obtain. Biobanks are essential in this regard for the discovery of novel predictive features.
2. Preventive: In order to stop the progression of the disease, biobanks-assisted advancement can use the prognostic value of early symptoms and combine them with genetic data to make an accurate diagnosis quickly and to give the appropriate therapy at the appropriate time.
3. Personalized: The effectiveness of treatment is greatly impacted by variations in the genotype and phenotype of the human population. Thorough understanding of the patient's genetic makeup and environmental factors improves diagnosis and therapy accuracy.
4. Participatory: P4 medicine is built on patients' and doctors' growing knowledge of each other and their mutual communication. As a result, IT solutions are essential for processing and managing the massive volumes of patient data that are gathered.

Time magazine listed biobanks as one of the “10 Ideas Changing the World Right Now” in 2009 (10), and for good reason—biobanks allow scientists to extract knowledge from thousands of samples. Solutions in personalized medicine are beneficial for oncologic illnesses (6). Cancer-oriented biobanks are a long-term source of human biological samples with associated data, collected at the time of diagnosis and during subsequent therapeutic phases. They are based on the collection of biological samples from patients with a specific disease (cancer) and controls, i.e., healthy tissues from cancer patients (11). “Organized databases of medical images, and associated imaging biomarkers shared among multiple researchers, linked to other biorepositories” is how the Imaging Biobanks working group of the Research Committee, which was formed by the European Society of Radiology, defines this novel field (12). These are not biological sample collections, in contrast to “traditional” biobanks. Instead of being just a list of bioimages linked to additional patient clinical data, imaging biobanks use cutting-edge computer technologies that allow image data, metadata, and raw data to be used for imaging measurements and biomarker extrapolation (13, 14). This allows radiomics and radiogenomics to be implemented, improving patient outcomes in the process.

Imaging biobanks have grown because of the vast amount of data and the need to gather it in a systematic and goal-oriented manner. This is also related to high-throughput computing's capacity to extract a wide range of quantitative information from bioimages created using cutting-edge CT, MR, and PET techniques (15, 16). The evaluation of extracted traits, pathological processes, and pharmacological reactions to a treatment intervention are the main objectives of radiomics (17).

Naturally, genomics dates back to the late 1980s, therefore radiomics is not the first “omics” discipline (18). Regarding radiomics, the intention is to use statistical and mathematical methods to the data found in the medical imaging. Thus, radiomics is a quantitative method of approaching medical imaging that adds to the data that physicians already have access to by applying sophisticated mathematical analysis. By analytically determining the spatial distribution of signal intensities and pixel inter-relationships, radiomics analyzes textural information.Numerous imaging studies from various fields have already been published using this methodology. Neoplastic pathology is one of radiomics' most often used applications. This is made possible by describing the pixel gray level distribution patterns, which machine learning (ML) systems may then analyze and potentially provide details about tumor physiology. This information may have a major impact on how these malignancies are managed and may soon lead to an improvement in their prognosis (19).

“Biobanks (which focus only on the collection of genotype data) should come with a system to collect related clinical or phenotype data,” states the European Society of Radiology (12). In order to guarantee approved technological validation, transparent sharing of biological and clinical data, and standardization of data collection and analysis, biobanks are essential (13).

According to Bonmatí et al. (20), these processes are essential for a successful translation of an imaging biobank into clinical practice. They emphasize that clinical validation acts as a bottleneck, separating valuable biobanks from useless ones. The oncologic community in particular is becoming more and more conscious of the significance of imaging biobanks (21). The purpose of our paper is to present the current state of the art regarding imaging biobanks, emphasizing their advantages and disadvantages over conventional biobanks as well as the ethical and legal issues that the scientific community constantly faces in the lack of a clear regulatory framework.

## 2 Methods

We conducted a thorough literature research on this subject by looking through earlier published articles. We conducted a literature search on Scopus (https://www.scopus.com), PubMed (https://pubmed.ncbi.nlm.nih.gov), and Google Scholar (https://scholar.google.com) using the following keywords: “Biobank” AND “Imaging” AND/OR “ethics” AND/OR “legal” AND/OR “statement” AND/OR “legislation” AND/OR “informed consent”]. Literature review results using PubMed database are shown in Table 1.

**Table 1:** Literature review results using the pubMed database.

Keywords	Search mode	Results
Imaging and biobank or biobanking	Imaging [Text Word] AND (biobank [Text Word] OR biobanking [Text Word])	1,250
Imaging biobank	Imaging biobank [Text Word]	6 [12, 13, 22–24, 35]
Imaging biobanks and ethics	imaging biobank [Text Word] AND ethics [Text Word]	1 [13]
Imaging and biobanks and legal or law or legislation	Imaging biobank [Text Word] AND law [Text Word] OR legislation [Text Word]	0
Imaging and biobanks and informed consent	Imaging biobank [Text Word] AND informed consent [Text Word]	1 [12]
Imaging and biobanks and statement	Imaging biobank [Text Word] AND statement [Text Word]	0

## 3 The imaging and “traditional” biological biobanks

The study's findings indicate that although interest in imaging biobanks is growing (22–24) and the scientific community is putting more effort into studying classical biobanks, there are still few investigations on the regulatory side of things.

To correlate patient clinical data with established biological biomarkers, imaging and “traditional” biological biobanks must be connected (25). Moreover, method harmonization is necessary to ensure that the features collected may be repeated and that imaging biobanks can be used in a variety of diagnostic scenarios (20). To evaluate the value of imaging biobanks in a therapeutic context, these actions are essential. One could view imaging biobanks connected to biological samples and clinical data about patients as a new frontier in biobanking. They might result in the creation of multi-omics biobanks, where genomic, proteomics, or metabolomics data would be combined with radiomic data to provide a novel and individualized method of treating disease (25).

Clinical outcomes are impacted by decisions made using imaging and other “omics” data; therefore, there is no justification for restricting the capabilities of these algorithms. This strategy is comparable to clinical practice, in which the decision-making process combines all patient data that is currently accessible with prior information from other situations. Thus, to increase diagnostic accuracy, radiomics-based algorithms may use immunomics, genomes, or other clinical data. As part of a bold paradigm change, radiomics may also be incorporated as a part of an all-encompassing software system for clinical decision support that combines clinical data and all “omics” into a “holomics” approach (the word “holo” in classical Greek means “whole”), much like systems biology in experimental research (26, 27).

Radiomics, in particular, has evolved from its early emphasis on prognosis (i.e., evaluating disease but not therapy) and lesion identification (e.g., malignant vs. non-malignant lesions). More recent algorithms incorporate genetic or immunomic characteristics to improve prediction of clinical outcomes (e.g., overall survival or toxicity) and to address treatment selection or response (i.e., predictive rather than prognostic) (28, 29). These methods are precisely referred to as radiogenomics and radioimmunomics, respectively. Moreover, radiomics is one of the “omics” axes utilized for clinical management in precision medicine techniques, and the term “holomics” is used to describe more ambitious approaches (30).

In order to mediate a change in medical practice and patient management, the medical imaging community could take the lead in this transformation towards precision medicine based on complete holomics computer-assisted expert systems (31). By employing quantitative data obtained from many digital imaging sources, modern radiology and nuclear medicine can really provide multiple imaging biomarkers of the same patient (12).

## 4 Focus on Italy

Europe has the majority of imaging biobanks (14). The European Commission established the Biobanking and Biomolecular Resources Research Infrastructure Consortium in 2013 (32). It is one of the biggest biobanking research infrastructures in Europe, presently comprising 23 nations and one international organization (32). There are numerous biobanks focused on diseases in Italy, however there isn't a central database or information access point. The European Research Infrastructure of Biobanks and BioMolecular Resources (BBMRI-ERIC) comprising 25 nations, 20 full members and 5 observers (33) has a National Node in Italy.

The Ministry of University and Research and the Ministry of Health collaborated to establish it. The Superior Institute of Health, the National Research Council, Scientific Hospitals and Treatment Institutes (IRCCS), universities, hospitals, researchers, and university research groups are examples of research institutions. Furthermore, a network of partners, comprising scientific societies, biomedical and biotechnology firms, and patient associations, supports and works in tandem with the node to establish goals and offer knowledge. Biobanks, Biological Resource Centers, and Collections spread throughout many Italian regions, along with three Common Services (CS Quality Management, CS Information Technology, and CS ELSI for ethical, legal, and social issues) comprise the statewide infrastructure known as BBMRI.it. The website provides easy access to the researchers' contacts.

In Italy, the laws controlling the processing of personal data, especially genetic data, are intimately related to those regulating research on biological samples and, consequently, biobanks. “Provisions for the adaptation of national legislation to the provisions of Regulation (EU) 2016/679 of the European Parliament and of the Council of 27 April 2016 concerning the protection of individuals with regard to the processing of personal data, as well as the free circulation of such data and repealing Directive 95/46/EC” is the Legislative Decree 10 August 2018 (34), n. 101, which establishes the legal framework.

NAVIGATOR (35), an Italian regional project aimed at developing an open imaging biobank for the collection and preservation of a large amount of standardized imaging multimodal datasets (CT, MRI, PET) tomography data, along with the corresponding patient-related and omics-related relevant information extracted from regional healthcare services or UK biobank and its several applications (36, 37), are some of the exemplary cases that already constitute a solid basis on which to work. Another noteworthy exception is the National German Cohort (38), a biobank including diagnostic imaging. According to Dagher (39), there are particular regulations pertaining to biobanks in several nations.

In addition to this particular national law, biobanking activities are subject to unique requirements in Australia, Ireland, France, Germany, Italy, Japan, and Switzerland. Without making reference to the recently defined concept of biobanks, all of these rules and recommendations were developed to control the establishment and operation of “traditional” biobanks.

## 5 Ethical-legal reflections and operational limits

While the creation of biobanks was an important milestone in the history of medical research, it is important to recognize that their growth has been decentralized. The different national mandates imposed by local government (data protection legislation) (40) and, from a technological standpoint, the diverse methodologies for collecting, storing, and validating basic data (41) have resulted in a significant degree of variance within biobanks. The main goal of an international research framework that aims to increase access to human biological resources may be hampered by these factors. The life cycle phases, which are intended to include the collection, accession, acquisition, identification, preservation, long-term storage, quality control (QC), transit, and disposal of biomaterials, are one of the primary sources of variability in biosamples (13).

Similar to biosamples in the early days of traditional biobanking, medical photographs were first either underrepresented or not collected at all in biobanks (42). Standardization/harmonization and validation of at least minimal rules for access and reimbursement are necessary in order to facilitate the broad and efficient use of human biological material (43). In order to provide precise biological interpretation for data analysis and interpretation, sample collection methods need to be established, confirmed, and validated. In 2012, the US National Cancer Human Biobank published the first standard operating procedures (SOPs) for biobanks (44), which was a major step in the right direction.

They continue to be a model for biobanks worldwide, and they were created with the understanding that the lack of consistent, high-quality biosamples has impeded the progress of clinical research. Enhancing national and international biobanking procedures as a result of the implementation of these specialized approaches continues to be one of the main goals of biobanks worldwide. ISO 20387 states that biobanks can implement common harmonizing procedures for the processing and organization of biological samples in order to meet minimal standardization criteria. The ISO 20387:2018 standard attempts to provide verified biological material that can guarantee the reproducibility and comparability of scientific research results by controlling the life cycle phases of the biological materials.

With regard to the entire life cycle of data associated with them, from collection to storage, reception and distribution, transport and traceability, preparation and preservation, process quality control, and method validation and verification, its detailed instructions provide accurate tools for processes and procedures. It is imperative to establish uniform protocols for quality assessment, consent, sample collection, storage, and authorization. The result is clear: if more precise, high-quality samples are made available, research will advance and impact the provision of healthcare.

It seems clear that an optimal organization of biobanks represents very fertile ground for the development of artificial intelligence in the medical and health care fields.

The field of computer science known as artificial intelligence (AI) focuses on developing algorithms and systems that, via data processing and machine learning, can replicate human behavior. It is helpful to distinguish between machine learning and deep learning when it comes to learning and data processing (45). Within the field of artificial intelligence, machine learning focuses on creating algorithms and models that possess the ability to “learn” from data and past experiences, enabling them to perform better in particular tasks. In this machine learning process, patterns and relationships are found in the data by an algorithm, which then uses this knowledge to forecast and make judgments about fresh data.

A subfield of machine learning and artificial intelligence known as “deep learning” makes use of multilayered artificial neural networks to teach them how to recognize and classify images and other complicated data representation patterns. In deep learning, artificial neural networks are made up of many layers of parallel processing units, or neurons, that learn data features in a stepwise manner, beginning with the most basic representations and working their way up to the most complex. Multilayer artificial neural networks need inputs, which the algorithm subsequently converts into outputs. It goes without saying that purer and uniform the inputs, the more precise and specific the results will be (46). Therefore, it is imperative that the data be standardized and confirmed in this scenario if the inputs are provided by a biobank (for example, for the study of a particular neoplasm). This is the typical outcome of supervised learning (47). The term “supervised learning” really refers to a machine learning technique wherein a model is trained using a set of input data (referred to as “features”) and the matching output labels that have undergone prior annotation, or “supervised.”

In order for the model to generalize and make precise predictions about novel input data that has never been seen before, it must learn a function that maps inputs to the correct output labels. Put differently, the model is given a known input and a known response (output label), and it attempts to create a map between the two in order to use the knowledge it has gained from the training dataset to accurately predict new data. Unsupervised learning is an additional approach to machine learning wherein the model is trained on a collection of input data without the associated output labels (48).

Without any outside direction or oversight, the model in this sort of learning attempts to find patterns, correlations, and structures in the input data. Put differently, the model is given a collection of unlabeled input data and, in the absence of any prior labeling information, it finds comparable patterns or clusters of data (49). Last but not least, reinforcement learning requires the agent to learn a behavior policy through experimentation and exploration of the environment, in contrast to supervised and unsupervised learning, in which the model receives a set of input data and attempts to learn a function that maps inputs to output labels or tries to identify patterns in the input data (19, 50).

There can be issues if imaging collections are also referred to as biobanks. It seems essential for the advancement of research that imaging biobanks be included in larger biobank groups connected to other biorepositories (12, 14). Debatable topics like data ownership and informed consent apply to imaging biobanks as well, and this is primarily dependent on how integrated they are with “traditional” biobanks—those that gather biological, tangible specimens.

The results of an online survey conducted among researchers and biobankers (51) indicated the following: improved informed consent procedures, more inclusive and transparent biobanking, the role of biobanks in sharing samples and data with industry partners and foreign countries, and the need for real, practical, and hands-on ethical and legal guidance. The breakdown into groups (52) provides a condensed summary of the problems associated with the use of these new entities (6). In terms of ownership, precise rights or their denial should be determined from the outset of a biobank (6), and the use of specimens after a donor's death and sample transfers between international researchers seem to be contentious issues (53).

**Figure 1:**
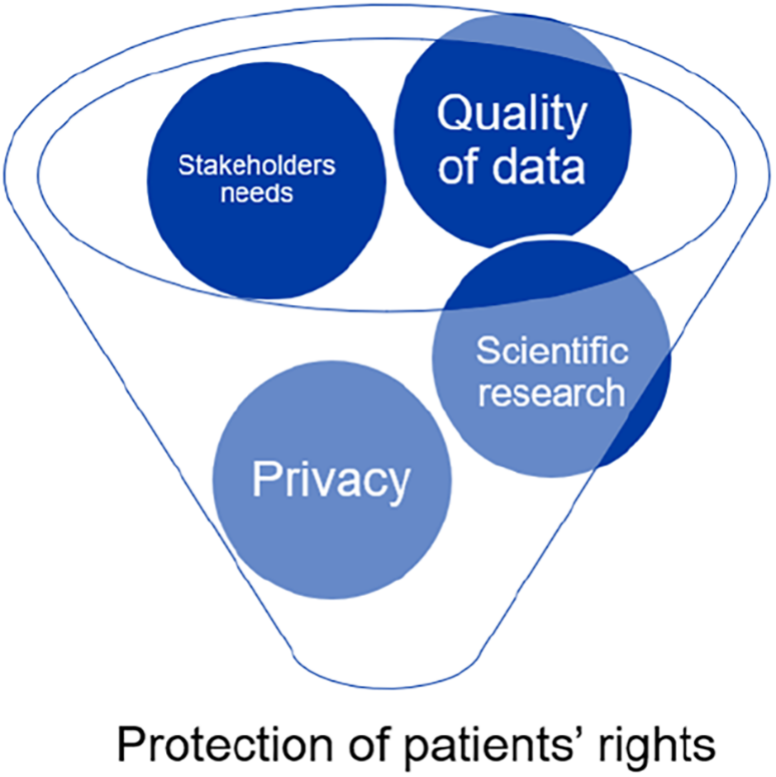
Biobanking activity: values involved in protection of patients’ rights.

The preservation of patients' rights is at the center of all factors related to biobanks, from the caliber of research to informed consent and privacy (see Figure 1).

It has been observed that in conventional biobanks, the patient would not be able to benefit from any future biotechnological study if the biological material belonged to an exclusive researcher, and that ownership of the biological sample is still up for question in Italy (54). In the particular instance of imaging biobanks, there are a few distinct ethical difficulties that need to be addressed. These issues may only be seen as partially overlapping and require independent attention.

Whereas the material dimension is paramount in traditional biobanks, the most significant and intricate aspect to oversee in imaging biobanks is the data dimension and its safety. In the age of digitization, ethical questions about sharing and storage are being questioned in a number of industries. However, these same worries take on even greater significance in relation to health data. Even if the creation of picture biobanks might offer patients more protection, there are still a lot of unanswered questions because there are no clear regulations or norms in this area.

The concept of informed consent restores a perfect balance between patients' right to self-determination and the autonomy of healthcare professionals, particularly researchers (55–60). Due to its limited validity to a specific purpose in a single study project, classical informed consent was inadequate from the outset of the establishment of traditional biobanks (51, 61, 62). As a result, a novel method of obtaining informed consent was developed, known as dynamic consent, which is a patient's consent to the use of his sample in both ongoing and future research as long as it is done so within the same parameters (6).

The patient must be approached again and their consent must be reapplied in the event that the framework changes (61). Pediatric biobanks present another contentious issue. Materials originating from minors may be stored in biobanks; in this instance, the child's will must be taken into consideration in addition to the approval of the parents or legal guardians (63). For instance, there are no particular laws governing pediatric biobanking in Italy (64).

Current legal measures should be tailored to the particular sector of biobanking, as there are currently no systematic international or national regulations that particularly address this matter (34). Italy is subject to the General Data Protection Regulation (EU) 2016/679 (GDPR), much like the rest of Europe (65). Therefore, only the issue of the processing of personal data can be deemed partially outdated in light of this legislation from an ethical analysis standpoint. While traditional biobanks with a longer history are still debating some ethical problems, imaging biobanks are in unknown territory with the European GDPR serving as the only point of reference.

Since the advent of image biobanks, ownership of the image has become a moral issue that needs to be resolved. The scientific community should investigate this new legal status further, particularly with regard to ownership, regulation, sharing, and data preservation. One can undervalue the fact that radiological images themselves make up a “biological sample” and that all current disciplines apply to it since images lack a materiality dimension. Informed consent, the freedom to “donate” one's image to an image biobank (without it being taken, anonymized, and combined into an image collection), the sharing of images between research centers, and the use of images for future research are among the ethical considerations that must direct the scientific community in this new field.

Anonymization and pseudonymization are two more significant difficulties arising from storage and protection (66). However, the requirement to remove information in the event of a biobank closure does not appear to apply to imaging biobanks (67). Furthermore, a significant topic of discussion in scientific literature appears to stem from the fact that, as defined by the GDPR, photographs do not count as personal data unless they can be connected to the patient's personal information, even in cases where they have been pseudonymized (35).

Notwithstanding, biometric data are defined as follows in Article 4(1)(14) of the GDPR: “personal data obtained by specific technical processing relating to the physical, physiological, or behavioral characteristics of a natural person and enabling or confirming their unique identification, such as facial image or fingerprint data.” It appears that the notion of biometric data, a subset of personal data, fits the case of radiological images since the images yield a unique identification of the subject to which they belong through straightforward processes of superimposition, analogy, and comparison. Table 2 provides a brief comparison of imaging and standard biobanks.

**Table 2:** Current status about different categories of biobanking.

Issues	Biobank (traditional)	Imaging biobank
Informed consent	Specific consent required	GDPR Regulation in Europe
Ownership	Still debated	Not yet a topic addressed
Specimen transfer	Specific consent required	Not yet a topic addressed
Post-mortem utilisation	Still debated	Not yet a topic addressed

## 6 Conclusions

The future of biobanking, with its intricate interactions involving patient and citizen engagement, national and international institution governance, and medical research and translation into clinical practice, appears to be closely tied to this (13). The ability to access biobanks and imaging biobanks facilitates research into the fundamental mechanisms underlying disease and the creation of novel biomarkers and therapeutics (68). For biobanks to flourish and be sustainable in the future, harmonization and standardization are essential components (69).

Improved harmonization can produce significant outcomes, including the ability to create studies on large cohorts and sub-cohorts of patients, obtain an orderly and multicenter collection of data and samples, and create homogenous patient groups with very large and well-described case histories (70).

In Italy the only imaging biobank currently operating is BCU Imaging Biobank (BCU-IB), which is a non-profit organization dedicated to gathering, archiving, and consulting diagnostic images, derived descriptors, and clinical data in order to advance imaging science and create new avenues for illness diagnosis, treatment, and possibly prevention.

BCU-IB is a collection-based biorepository that is open to a wide range of diseases, anatomical locations, and imaging modalities. It is designed to archive enormous volumes of human body pictures, both healthy and unwell, from retrospective and prospective cohorts (71).

The archiving of diagnostic pictures, clinical reports, and demographic data enables researchers to find correlations between genetic variables and phenotypes derived from imaging and lifestyle. The biobank has been operational for numerous scientific research projects since its foundation in 2018. From these initiatives, collections of individuals with COVID19 pneumonia, neurological disorders, and cancer have been derived. BCU-IB has been a member of the BBMRI-ERIC biobank network since 2020, specifically the Italian node BBMRI. The other European image biobank projects (NAVIGATOR, EUCANIMAGE, INCISIVE, CHAIMELEON, PROCANCER-I, PRIMAGE, EUCANSHARE), some of which are still under development, differ in that they are large image collectors with the idea of generating a pan-European repository of medical images that can be used for ML-based training for various types of cancer or other diseases (72). It is believed that the unstoppable trend in the world of science is to no longer have national image biorepositories, but to create multi-center collaborations that can increase the accuracy of diagnosis methods. It should come as no surprise that there are many biobanks with an oncologic focus because oncology has always been one of the professions that has benefited from biobank support (14). Innovations in artificial intelligence (AI) for pathology decision assistance, particularly in cancer, have quickly trailed the rise of digital pathology with the goal of optimizing and enhancing diagnostic pipelines (73). Should “traditional” biobanks include imaging biobanks in order to create a more comprehensive and expansive knowledge network? Since protocols and quality controls for biobanks have been thoroughly examined and analyzed (53, 74), the scientific community still needs to resolve a number of methodological issues, either with regard to the autonomy of imaging biobanks or the integration of imaging repositories into conventional biobanks that are disease-focused. While the potential of digital repositories has recently been explored (75), and comprehensive evaluations have been carried out in the field of imaging biobanks (42), ethical and legal considerations have not yet been properly addressed. A number of unanswered problems exist for researchers using biobanks in the absence of regulation similar to that for clinical trials: Can photos qualify as personal data under the GDPR? Is it possible to identify a patient uniquely using a radiological or radiometabolic image? What are the consent's validity bounds in relation to a novel study protocol? What are the restrictions on the validity of consent for processing data in order to conduct a new study? How is complete anonymization of the sample accomplished in the event that an imaging biobank is integrated into a conventional biobank? Due to the general inclination to want to establish scientific exceptions to the field of biobank imaging, all of these concerns—which the investigators have quite rightly raised—have not yet been addressed in the absence of a clear legal framework. It appears that biobanking is an important subject in which public-private partnerships, medico-legal ramifications, and imaging community requirements (76) need to be thoroughly considered and examined. However, for this integration to occur, a framework must be in place where questions about sample-data ownership, consent to experimentation, consent to data processing and potential revocation, restrictions on the use of samples for secondary purposes, and accountability for the proper storage, transfer, and sharing of data are first addressed using medico-legal expertise. A comprehensive definition of modern biobanking that fully meets the needs of the entire community is driven by radiology and nuclear medicine, as evidenced by the wealth obtained from imaging biomarkers, particularly in oncology. From a scientific perspective, “image collections” are acknowledged for their significance.

A further stage that necessitates an assessment of preliminary requirements (ex-ante evaluation) and expertise for data processing in compliance with legal and transparent criteria is the transition to an image biobank. This setup should ideally occur in a way that is harmonic and integrated with other “biorepositories,” such as conventional (i.e., tissue) biobanks. A lack of regulation is evident from the evaluation, pertaining to certain parts of traditional biobanks that are still up for controversy as well as a more general absence of regulatory framework for biobank imaging.

Legislative action at the European and global levels is needed to make clear the important issues that are only covered by “soft law” mechanisms. This final stage can result in a true standardization of data, clinical validation for rapid treatment use, and make it easier for the scientific community to use the data for study.
